# Novel glycoprotein SBSPON suppressed bladder cancer through the AKT signal pathway by inhibiting HSPA5 membrane translocation

**DOI:** 10.7150/ijbs.109973

**Published:** 2025-07-11

**Authors:** Beibei Ni, Shi Li, Lan Zhao, Lin Gao, Liya Luo, Junwen Zhang, Xina Xie, Yuqi Zhu, Wei Yang, Shasha Min, Yan Wang, Xianxin Li, Zhiming Cai, John R. Speakman, Zesong Li

**Affiliations:** 1 Guangdong Provincial Key Laboratory of Systems Biology and Synthetic Biology for Urogenital Tumors, Shenzhen Key Laboratory of Genitourinary Tumor, Department of Urology, Shenzhen Institute of Translational Medicine, The First Affiliated Hospital of Shenzhen University, Shenzhen Second People's Hospital, Shenzhen 518035, China.; 2 Vaccine Research Institute, Cell-Gene Therapy Translational Medicine Research Centre, The Third Affiliated Hospital of Sun Yat-sen University, Guangzhou 510630, China.; 3 Department of Medical Research Center, Yuebei People's Hospital Affiliated to Shantou University Medical College, Shaoguan 512026, China.; 4 Guangzhou National Laboratory, Guangzhou 510005, China.; 5 The Fourth Affiliated Hospital of Guangzhou Medical University, Guangzhou 511300, China.; 6 Department of Urology, Shenzhen Qianhai Taikang Hospital, Shenzhen 518000, China.; 7 Shenzhen Key Laboratory of Metabolic Health, Center for Energy Metabolism and Reproduction, Shenzhen Institute of Advanced Technology, Chinese Academy of Sciences, Shenzhen 518055, China.; 8 State Key Laboratory of Molecular Developmental Biology, Institute of Genetics and Developmental Biology, Chinese Academy of Sciences, Beijing 100101, China.; 9 Institute of Biological and Environmental Sciences, University of Aberdeen, Aberdeen, UK.; 10 China Medical University, Shenyang, Liaoning, China.; 11 Institute of Basic Medicine and Forensic Medicine, North Sichuan Medical College, Nanchong 637199, China.; 12 Institute of Future Agriculture, Northwest Agriculture and Forestry University, Yangling, Shanxi 712100, China.

**Keywords:** SBSPON, Bladder cancer, AKT signal pathway, HSPA5, Cisplatin resistance

## Abstract

Bladder cancer poses severe threats to human health due to its aggressive nature and resistance to drug treatment; however, the underlying mechanisms have not been fully investigated. In the present study, we identify SBSPON (Somatomedin B and Thrombospondin Type 1 Domain Containing) as a novel tumor suppressor. The expression of SBSPON was downregulated in bladder cancer and correlated with poor overall survival. SBSPON suppressed the proliferation and migration ability of bladder cancer cells* in vitro*, and inhibited tumor growth of bladder cancer cells *in vivo*. Genetic ablation of *Sbspon* in mice significantly accelerated the progression of N-butyl-N-(4-hydroxybutyl)-nitrosamine (BBN) induced bladder cancer. Mechanistically, SBSPON is a novel HSPA5 binding glycoprotein. SBSPON functioned through binding to HSPA5 and inhibiting its membrane translocation, resulting in an inactivated AKT signaling pathway. More importantly, SBSPON inhibited the cisplatin resistance of bladder cancer cells by reducing the inhibitory effect of HSPA5 on apoptosis. In summary, the novel glycoprotein SBSPON functions as a tumor suppressor and inhibits resistance to cisplatin in bladder cancer. This may provide novel therapeutic strategies for bladder cancer treatment.

## Introduction

Bladder cancer is a global health burden, with 613,791 estimated new cases and 220,349 estimated deaths worldwide in 2022^1^. Despite significant improvements in the management of bladder cancer including surgical interventions, chemotherapy, radiotherapy and immunotherapy, there are still challenges due to its high metastatic nature and development of drug resistance^2^. Cisplatin (DDP) is a first-line chemotherapy drug used for the treatment of bladder cancer, yet most patients quickly develop drug resistance to it^3^. With the advancement of precision medicine, cancer sub-classification based on mutations, biomarkers or genotype has become a valuable guidance for cancer treatment selection and prognosis^4^. Therefore, it is of significant importance to uncover novel functional molecular factors for the diagnostic and prognostic evaluation, and explore new mechanisms of drug resistance in bladder cancer^5^.

SBSPON, also known as Chromosome 8 Open Reading Frame 84 (C8orf84) or Retinal Pigment Epithelium-Spondin Protein (RPESP) was cloned in 2002^6^ and 2004^7^, respectively. The human SBSPON gene is located on chromosome 8, with 59,546 bases including 5 exons and 4 introns. This transcript length of SBSPON is 3,801 bps (NM_153225.4), encoding a protein consisting of 264 amino acids with 29.6 kDa predicted molecular mass (NP_694957.3). The SBSPON gene is evolutionarily conserved in mammals, including humans, chimpanzees, dogs, cattle, mice, and rats (Fig. S1A-B), which suggest that SBSPON may have important physiological functions. However, there are no reports on the expression and physiological function of SBSPON.

HSPA5 is a key unfolded protein response (UPR) regulator that normally resides in the endoplasmic reticulum (ER), where is involved in multiple cellular processes^8^-^11^. HSPA5 expression was significantly upregulated in most cancer types^12^-^14^. In the progression of cancer, HSPA5 is translocated to the cell surface (csHSPA5) where it activates key signaling pathways, such as the PI3K/AKT and MAPK/ERK signaling pathways, which promote cell survival and inhibit apoptosis^14^-^19^. In addition, HSPA5 is significantly involved in chemotherapy resistance in diverse cancer types^20^-^25^. HSPA5 is upregulated in bladder cancer and facilitates the progression of bladder cancer^12^, ^26^. However, the roles and underlying mechanisms of HSPA5 in bladder cancer remain unclear.

In the present study, we identified SBSPON as a novel HSPA5 binding glycoprotein and tumor suppressor, and investigated the molecular mechanism of its cellular function and regulatory roles in bladder cancer progression and cisplatin resistance. Our findings highlight the potential of SBSPON as a potential biomarker and therapeutic target, and provide new insights into tumor progression and resistance to cisplatin in bladder cancer.

## Materials and Methods

### Study design

The objective of this study was to elucidate the function of SBSPON in bladder cancer and to explore novel therapeutic strategies for its treatment. The mRNA and protein levels of SBSPON were assessed in bladder cancer patients, bladder cancer cells and a BBN-induced mouse bladder cancer model. The relationship between SBSPON, tumor stage, metastases, grade and survival in bladder cancer were assessed. The functional gain and loss experiments of SBSPON were also performed both *in vitro* (different types of bladder cancer cells) and *in vivo* (nude mice and C57BL/6J). The mice were fed a chow diet, provided with sterile drinking water ad libitum, and maintained on a 12-hour light/dark cycle. Differences in expression of genes, which encoding different cell signaling molecules that regulate cell proliferation, apoptosis, migration, invasion and tumorigenesis, were examined. Detailed functional sites characterization of SBSPON were also performed by point mutations. The interacting partners of SBSPON were examined by Co-immunoprecipitation. The cell experiments were repeated at least three times. The mice were used with a minimum of five mice in each experimental group and mice were randomized to groups.

### Clinical specimen collection

The bladder cancer and adjacent noncancerous tissues were obtained by tissue biopsy from patients diagnosed with bladder cancer at the Shenzhen Second People's Hospital between March 2011 and September 2015. Tissue microarrays were provided by Shanghai Outdo Biotech (Shanghai, China). The studies using human tissue were approved by the Ethics Committee of Shenzhen Second People's Hospital (approval no. KS201900604004) and the Ethics Committee of Shanghai outdo Biotech Company (approval no. YB M-05-02). Informed consent was obtained from patients. All diagnoses of bladder cancer were histopathologically confirmed. Clinicopathological characteristics (including age, sex, clinical stage, lymph node metastasis status and pathological grade) were obtained from medical records and pathology reports.

### Western blot analysis

Cell and tissue extracts (30-50 μg per lane) were prepared in lysis buffer (10 mM Tris-HCl, 150mM NaCl and 1% Triton X-100) containing protease inhibitors, and then subjected to western blotting. The western blot was probed with the following antibodies: antibodies against E-cadherin (catalog #31020), N-cadherin (catalog #7939), Vimentin (catalog #7557), p-ERK1/2 (Tyr202/Tyr204, catalog #16982), MEK (catalog #436), and p-MEK (catalog #7995) were obtained from Santa Cruz Biotechnology Inc. Antibodies against AKT (catalog #9272), ERK1/2 (catalog #9102), p-AKT (Ser473, catalog #4060), GSK-3β (catalog #9832), p-GSK-3β (catalog #9322), Snail (catalog #3879), P27 (catalog #3686), P21 (catalog #2947) and β-catenin (catalog #8814) were obtained from Cell Signaling Technology. Antibodies against HSPA5 (catalog #21685), β-Tubulin (catalog #6046) and β-actin (catalog #8227) were obtained from Abcam. Antibodies against SBSPON (catalog #029595), Flag (catalog #7425) and Myc (catalog #2276) were obtained from Sigma-Aldrich. SBSPON (catalog #18938) was obtained from Proteintech. Protein band quantification was performed using LabImage software.

### Quantitative real-time PCR (qPCR)

RNA extraction from cells was performed using TRIzol Reagent (Invitrogen) following the manufacturer's instructions. Reverse transcription was conducted using ReverTra Ace qPCR RT Master Mix with gDNA Remover (Toyobo, Japan). QPCR was carried out with SYBR Green Real-time PCR Master Mix (Toyobo, Japan) with the following cycling conditions: an initial denaturation step at 95°C for 10 min; followed by 40 cycles of denaturation at 95°C for 15 sec; and annealing/extension at 60°C for 1 min. For each qPCR assay, the housekeeping gene encoding β-actin was used as an internal control. Triplicate qPCR reactions were performed, including no-template controls. The relative expression of SBSPON was determined using the comparative cycle threshold (CT)(2^-△△CT^) method. The forward and reverse PCR primers are listed as follows: SBSPON, 5'-CGGGCACACCTATGTTCCTG-3' and 5'-TCCATACAGTATCCAGCATCCTCT-3'; HSPA5, 5'-CATCACGCCGTCCTATGTCG-3' and 5'CGTCAAAGACCGTGTTCTCG-3'; and β-actin, 5'-TGAAGATCAAGATCATTGCTCCTC-3' and 5'-AACTAAGTCAT AGTCCGCCTAGAAG-3'.

### Cell culture and plasmids

TCCSUP, SW780, 5637, J82, T24 and UMUC3 were obtained from the American Type Culture Collection. Plasmids expressing SBSPON (tagged with 3×Flag) or HSPA5 (tagged with Myc) were constructed by in-frame insertion of human SBSPON or HSPA5 cDNA into the pcDNA3.1 expression vector. For construction of the SBSPON mutants, we performed PCR using the pcDNA3.1-3×Flag-SBSPON plasmid as template DNA. All mutations were verified by DNA sequencing.

### Lentiviral vector construction and siRNA transfection

The lentiviral plasmids pLVX-EGFP-3xFlag as empty vectors (control) or with SBSPON cDNA were constructed by Genechem (Shanghai, China). T24 and 5637 cells, with low detectable SBSPON expression, were subjected to lentiviral infection to generate stable cells. Cells were also infected with viruses containing the same amount of control vector. SW780 and UMUC3 cells were transfected with siRNA oligonucleotides using the Lipofectamine 3000 Transfection Reagent (Invitrogen, Carlsbad, CA, USA) in 6-well plates and incubated for 48 h. The SBSPON siRNA used in the experiments was synthesized by Invitrogen (Carlsbad, CA, USA). The following target sequences were used: Human SBSPON, 5'-GACAGAGTCCTTGACTCCTCACTGT-3'.

### Immunoprecipitation

Cell lysates from 5 × 10^6^cells were treated with NP-40 and incubated with antibody. Next, 30μl of prewashed protein A/G-conjugated agarose beads (Sigma-Aldrich) were added to the lysates. The beads were boiled and centrifuged. Subsequently, the proteins were separated using SDS-PAGE. The bands that interacted with SBSPON were subjected to peptide sequence analysis using mass spectrometry.

### Immunohistochemistry

The paraffin-embedded sections were subjected to deparaffinization using xylene, followed by rehydration through a series of decreasing ethanol concentrations. Antigen retrieval was performed by heating the sections in citrate buffer. The slides were incubated with a rabbit anti-SBSPON antibody at 4°C overnight. Anti-rabbit horseradish peroxidase-conjugated secondary antibodies were then applied. The slides were further incubated with streptavidin-horseradish peroxidase complex and diaminobenzidine (DAB). Hematoxylin was used for counterstaining, followed by dehydration and mounting. The slides were evaluated by a blinded and experienced pathologist who was unaware of the patient's condition. The staining intensity was assessed using a scale ranging from 0 to 3, while the heterogeneity of staining was evaluated on a scale of 0 to 4, based on the percentage of tumor cells showing positive staining. According to the receiver operating characteristic (ROC) analysis, a cumulative evaluation score (CES) of 7 or lower was considered indicative of low SBSPON expression.

### Cell viability assay and colony formation assay

To evaluate the proliferative capacity of cells, a Cell Counting Kit 8 (CCK-8) from Dojindo Molecular Technologies, Inc. (Kumamoto, Japan) was utilized. Cells were seeded on 96-well plates. At specified time intervals, 10µl of CCK-8 solution was added and incubated. The absorbance was measured at 450 nm using a Multiskan Go instrument from Thermo Scientific (MA, USA). For colony formation assay, cells were seeded in plates and fixed with 4% polyoxymethylene and stained with 0.05% crystal violet solution. Colonies containing more than 50 cells were counted. Each experiment was repeated three times with three technical replicates.

### Cell migration and invasion assays

The cell invasion assays were conducted using an insert plate with a chamber matrigel-coated membrane (BD Bioscience, MA, USA). Cells were placed in the upper chambers of the Transwell plates, and 20% FBS-DMEM was placed into the basal chambers as a chemoattractant. After the plate was incubated for 48 h, the invaded cells were fixed using a solution of 4% paraformaldehyde. Then the cells were stained with 0.05% crystal violet solution. Cell migration assays followed the same protocol but without Matrigel on the inserts. Each experiment was repeated three times with three technical replicates.

### Cell cycle and apoptosis assays

For cell cycle analysis, cells were stained with a cell cycle staining solution obtained from BD Biosciences. Flow cytometry was used to analyze the stained cells. Specifically, the cells were incubated with 1 ml of DNA staining solution and sorted using a FACSCalibur instrument from BD Biosciences (Franklin Lakes, NJ, USA). To detect cell apoptosis, an Annexin V-FITC/PI apoptosis detection kit from BD Biosciences was employed. For this assay, Annexin V-FITC and PI were added to the cells, which were then incubated for 15 minutes in the dark. The cells were subsequently examined using flow cytometry.

### Xenograft tumor assay

Male nude mice (BALB/c-nu) of four-week-old, weighting 16-19 g, were purchased from Guangdong GemPharmatech Co.,Ltd. The animal experiments conducted in this study were approved by the Animal Ethics Committee of the China Technology Industry Holdings (Shenzhen) Co., Ltd. Cells were injected into mice. At the end of the experiments, the immunodeficient mice were euthanized. The tumor tissues were weighed, measured, and prepared for immunohistochemical staining analyses. Tumor volume was calculated with the formula: (length×width^2^)/2.

### Generation of SBSPON transgenic mice and BBN treatment

The Model Animal Research Center (Nanjing University, China) generated SBSPON knockout mice (C57BL/6J) using the CRISPR/Cas9 system. The gene-specific gRNA targeting the SBSPON gene was designed to induce a double-strand break (DSB) through direct Cas9 endonuclease cleavage. The DSB was repaired through the non-homologous end joining (NHEJ) pathway, resulting in the deletion of exon 2. Therefore, the reading frame of the SBSPON gene was disrupted due to a frameshift deletion that resulted in loss of the protein encoded by SBSPON or a truncated protein. *Sbspon^-/-^* mice and their wild-type counterparts (*Sbspon^+/+^*) were identified by PCR and sequencing. SBSPON knockout mice bred normally and survived to adulthood. These mice were aged and maintained until death. The construction of SBSPON-knockout mice was carried out by the Model Animal Research Center (Nanjing, China). The animal experiments were approved by the Animal Ethics Committee of Shenzhen PKU-HKUST Medical Center. BBN was purchased from Shanghai TCI Company. Heterozygous and homozygous SBSPON knockout mice, aged 6 to 8 weeks, were randomly assigned to two groups: a BBN-treated *Sbspon^+/+^* mice group (n = 7) and a BBN-treated *Sbspon^-/-^* mice group (n = 7). Mice in the BBN-treated groups were supplied with ad libitum access to tap water containing 0.05% BBN for a duration of 18 weeks. The BBN solution was kept in dark-colored bottles. Drinking water was replaced twice weekly during the experimental period. The mice were euthanized at the conclusion of the experiment. The tissues were harvested, processed for paraffin embedding and sectioning, and stained with hematoxylin and eosin.

### Immunofluorescence and confocal microscopy

Cells plated onto glass coverslips were fixed in 4% paraformaldehyde (PFA) and permeabilized with 0.1% Triton X-100 in PBS. Primary antibody incubations were carried out at 4°C overnight. Coverslips were incubated with the Alexa Fluor-conjugated secondary antibody (Life Technologies). Nuclei were visualized with DAPI (Life Technologies). The slides were imaged with a Nikon 90i microscope using NIS Elements software, a Leica DM IRBE microscope using SimplePCI 6 software or a Zeiss LSM780 confocal microscope using Zeiss ZEN2011 software.

### TUNEL assay

Nuclear DNA fragmentation was quantified to assess apoptosis in both the control cells and drug-treated cells. The TUNEL Apoptosis Detection Kit (Alexa Fluor 640) (Yeasen; #40308ES20) was used for this purpose. Nuclei were counterstained with DAPI. Fluorescent images of apoptotic cells were captured using a LSM800 laser scanning confocal microscope (Carl Zeiss microscopy, Germany).

### Protein-Protein Interaction (PPI)

To predict interactions between SBSPON and HSPA5, the PPI prediction model was employed, which comprehensively captures and analyzes the complex interaction relationships between proteins^27^. PPI utilizes a one-dimensional convolutional neural network in deep learning technology to predict protein-protein binding sites. Inputting the primary sequences of SBSPON (amino acids 21-264) and HSPA5 (residues 19-654), the model calculates the binding propensity for each amino acid pair interaction.

### Protein-protein docking methodology

The structures of proteins SBSPON and HSPA5 were predicted using AlphaFold^28^, ^29^. The signal peptides were removed from both protein structures, specifically residues 1-20 for SBSPON and residues 1-18 for HSPA5. For protein-protein docking, we utilized MEGADOCK 4.0^30^, a molecular docking software based on the Fast Fourier Transform (FFT) algorithm. According to the prediction results from the PPI software, amino acids with binding propensity scores greater than 0 were selected as binding sites, with residues 75-125 of SBSPON designated as the primary interaction region. The docking simulation was performed under rigid-body conditions, with grid spacing and rotation step parameters of 1.2 Å and 15°, respectively. The conformation with the lowest binding score between SBSPON and HSPA5 was selected as the optimal complex structure. Protein-protein interactions were subsequently analyzed using PDBePISA 1.4.0, and the results were visualized using PyMOL 2.2.0.

### Statistical analysis

Statistical analyses were conducted using either the SPSS statistical package (Version 16.0, Chicago, USA) or GraphPad Prism 8.0 software (CA, USA). The data are presented as the means ± standard deviations (SDs) or standard error of the mean (SEM). Differences between groups were assessed using unpaired Student's t-tests, the Mann-Whitney U test or paired t-tests. To assess the associations between SBSPON expression or clinicopathologic parameters and overall survival, univariate and multivariate analyses were performed using a Cox proportional hazards model. The experiments were replicated at least three times. A significance level of *P* < 0.05 was used to determine statistical significance in all analyses. In the figures, *, ** and *** refer to *P* < 0.05, *P* < 0.01 and *P* < 0.001, respectively.

## Results

### The expression of SBSPON is downregulated in bladder cancer and correlates with poor prognosis of bladder cancer patients

**Figure 1:**
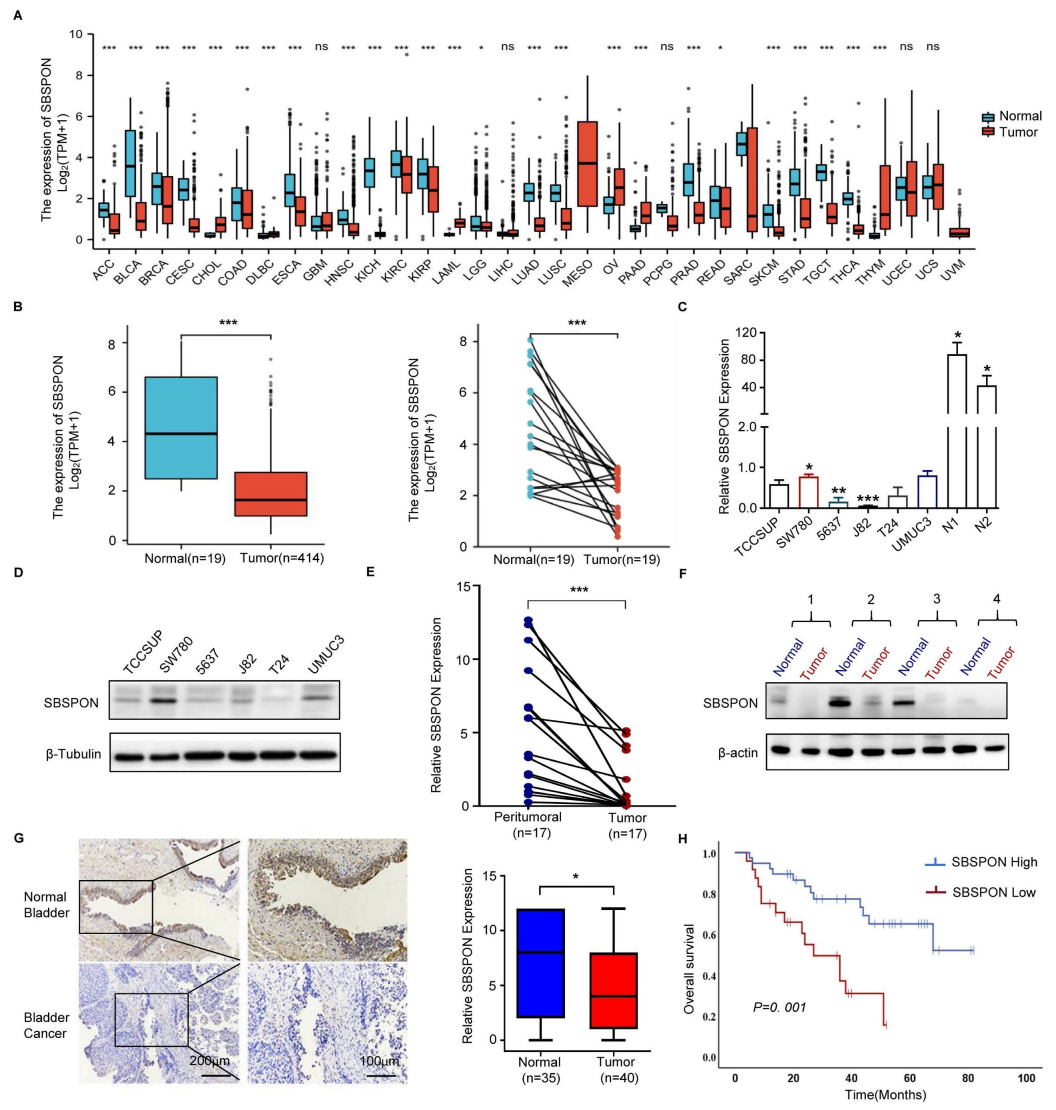
** Analysis of the clinical relevance of SBSPON expression in bladder cancer.** (A) SBSPON was down-regulated in 20 tumors from TCGA. (B) The analysis of TCGA data revealed that the expression of SBSPON was significantly lower in bladder cancer compared to normal bladder. (C and D) QPCR and western blot analysis were performed to assess the expression of SBSPON in bladder cancer cell lines. N1 and N2 indicate the two normal bladder tissues. β-actin and β-Tubulin were utilized as a loading control. (E and F) QPCR and western blot analysis were performed to assess the expression of SBSPON in bladder tumor tissues. (G) The expression of SBSPON was analyzed by immunohistology chemistry staining in healthy samples and bladder tumor tissues, as indicated. (H) Kaplan-Meier overall survival curves for bladder cancer patients with high or low SBSPON expression. *P < 0.05, **P < 0.01, ***P < 0.001.

To assess the potential role of SBSPON in bladder carcinoma, we first examined SBSPON expression using our previous transcriptome dataset^31^, ^32^. We found that the expression of SBSPON is downregulated in human bladder carcinoma tumors as compared with normal bladder tissues^31^, ^32^. Consistently, in TCGA database, SBSPON was significantly downregulated in various types of tumors as compared with normal tissues, including bladder cancer (Fig. 1A-B). We further validated these observations by experimentally measuring SBSPON levels in different tissues and cell lines. The results showed that SBSPON was widely expressed in most normal human tissues (Fig. S2A) while its expression was variable in different human tumor cell lines (Fig. S2B). However, the expression of SBSPON was markedly lower in bladder cancer cells (Fig. 1C-D), and significantly decreased in bladder tumor tissues as compared with adjacent normal tissues (Fig. 1E-F).

To understand the clinical relevance of SBSPON expression in bladder cancer, the relationship between SBSPON expression levels and the clinicopathological variables of bladder cancer patients was analyzed by using immunohistochemical staining on a bladder cancer high density tissue array and a bladder cancer survival tissue array with a validated antibody against SBSPON.

The results showed that SBSPON protein was mainly visible in normal bladder urothelial cells as intermediate to intense cytoplasmic staining, and significantly decreased in bladder tumor cells (Fig. 1G). SBSPON expression was significantly associated with clinical stage (*P* = 0.03), pathological grade (*P* = 0.023) and regional lymph node metastases (*P* = 0.013) (Table 1).

A Kaplan-Meier survival analysis was performed to assess the association between SBSPON levels and patient survival data based on 62 bladder cancer tissues with survival information. The results showed that decreased SBSPON expression was associated with poorer overall survival (*P*=0.001, Fig. 1H). Cox regression analysis was performed to identify whether the expression of SBSPON is an independent prognostic factor of bladder cancer. Univariate cox regression analysis demonstrated that the expression level of SBSPON (*P*=0.0016) and regional lymph node metastasis (*P*=0.0003) were predictive factors for prognosis (Table 2). Multivariate cox regression analysis demonstrated that the regional lymph node metastasis and the relative level of SBSPON expression was an independent prognostic factor for poor overall survival (*P* = 0.015 and *P* = 0.026, respectively) (Table 2).

### SBSPON inhibits cell proliferation, induces cell cycle arrest and promotes cell apoptosis in bladder cancer

**Figure 2:**
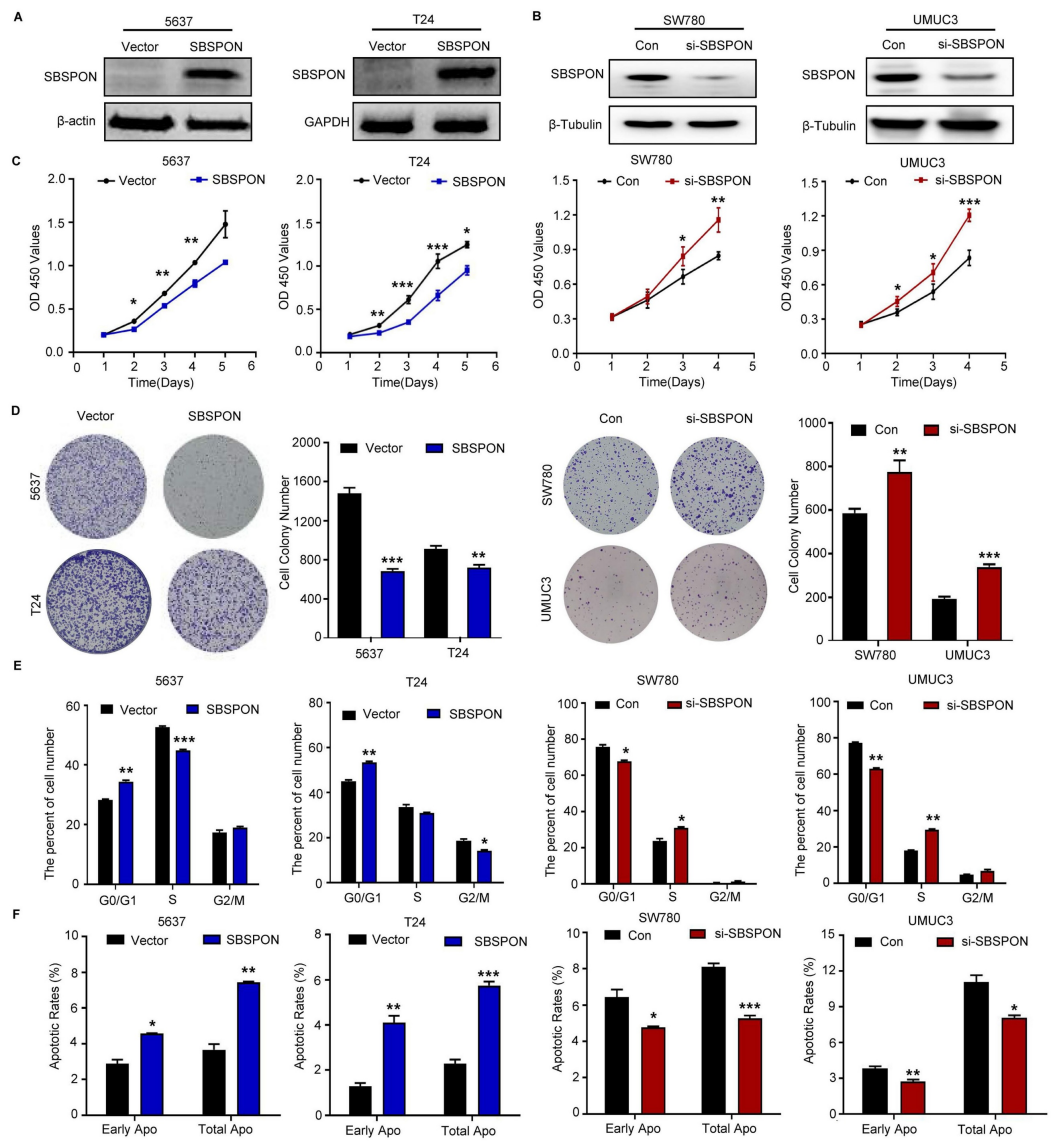
** SBSPON inhibits cell proliferation in bladder cancer.** SBSPON expression was determined by western blot analyses following transduction using lentiviral construct expressing SBSPON or control lentiviral construct. β-actin and GAPDH were utilized separately as loading controls. (B) Knockdown of SBSPON expression by *SBSPON*-specific siRNA was confirmed. β-Tubulin was utilized as a loading control. (C) The effect of SBSPON on the proliferation of bladder cancer cells was evaluated using CCK-8 assays. (D) The effect of SBSPON on the proliferation of bladder cancer cells was evaluated using colony formation assays. (E) The influence of SBSPON on the cell cycle was analyzed using flow cytometry. (F)The impact of SBSPON on the apoptosis was evaluated using flow cytometry. *P < 0.05, **P < 0.01, ***P < 0.001.

To explore the potential role of SBSPON in bladder carcinoma, we generated stable SBSPON-overexpressing 5637 and T24 bladder cancer cell lines using lentiviral vectors (Fig. 2A), and silenced SBSPON expression in SW780 and UMUC3 bladder cancer cell lines by transfecting siRNA (Fig. 2B). Cytological assays showed that compared with control cells, SBSPON overexpression significantly inhibited the proliferation and colony formation of 5637 or T24 cells, while SBSPON knockdown in SW780 and UMUC3 cells showed opposite effects (Fig. 2C-D).

Additionally, it was evident that SBSPON-overexpression arrested the growth of 5637 or T24 cells in the G0/G1 phase and increased the ratio of apoptosis cells based on flow cytometric assays of cell cycle and apoptosis (Fig. 2E-F, Fig. S2C-D). Conversely, the number of bladder cancer cells in the G0/G1 phase and the apoptotic rates were significantly decreased when SBSPON was silenced in SW780 and UMUC3 (Fig. 2E-F, Fig. S2C-D).

We next detected the effects of SBSPON overexpression on the expression of cell cycle regulators. The results showed that SBSPON overexpression significantly enhanced the levels of the G1 gatekeepers P27 and P21 (Fig. S2E). The results suggested that SBSPON induces cell cycle arrest at the G0/G1 checkpoint in bladder cancer cells.

### SBSPON inhibits cell migration, invasion and EMT in bladder cancer

**Figure 3:**
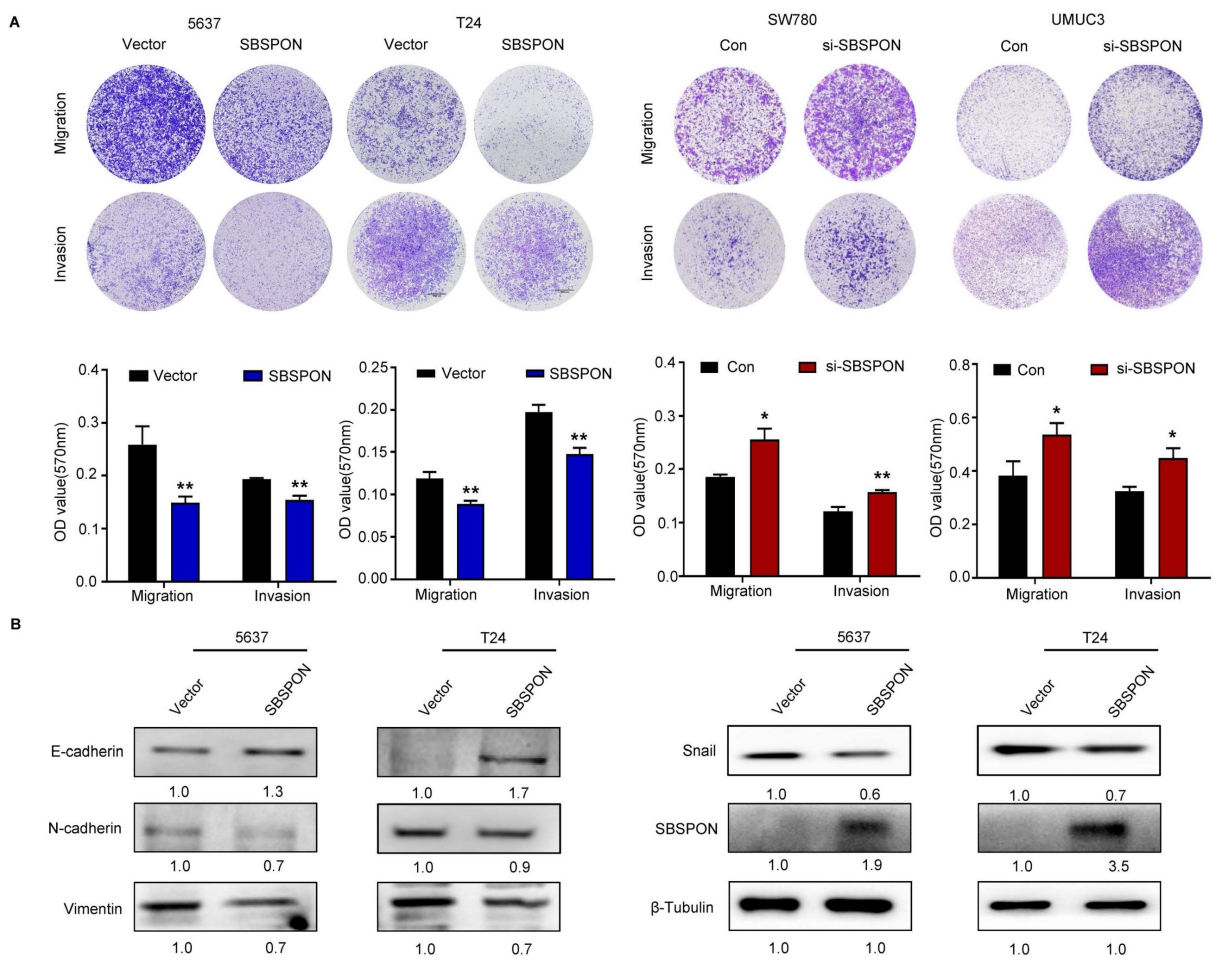
** SBSPON inhibits cell migration and invasion in bladder cancer.** The impact of SBSPON on cell migration and invasion were accessed by transwell assays. *P < 0.05, **P < 0.01. (B) Western blot analysis was performed to assess the levels of epithelial cell markers and mesenchymal cell markers in 5637 and T24 cells. β-Tubulin was utilized as a loading control.

Cell migration and invasion have been identified as key events in cancer development. Therefore, we investigated the effect of SBSPON on the migration and invasion of bladder cancer cells. Transwell assays showed that SBSPON overexpression significantly impaired the migration and invasion abilities of 5637 and T24 cells, while SBSPON knockdown in SW780 and UMUC3 cells showed opposite effects (Fig. 3A). To test if SBSPON inhibited migration and invasion by regulating the process of EMT, we assessed the effects of SBSPON overexpression on the expression levels of EMT-related proteins. The results showed that SBSPON overexpression induced an epithelial phenotype by reducing mesenchymal markers (Vimentin, N-cadherin and Snail) while increasing the epithelial marker E-cadherin (Fig. 3B), suggesting that SBSPON inhibits EMT in bladder cancer cells.

### SBSPON represses* in vivo* bladder tumorigenesis

**Figure 4:**
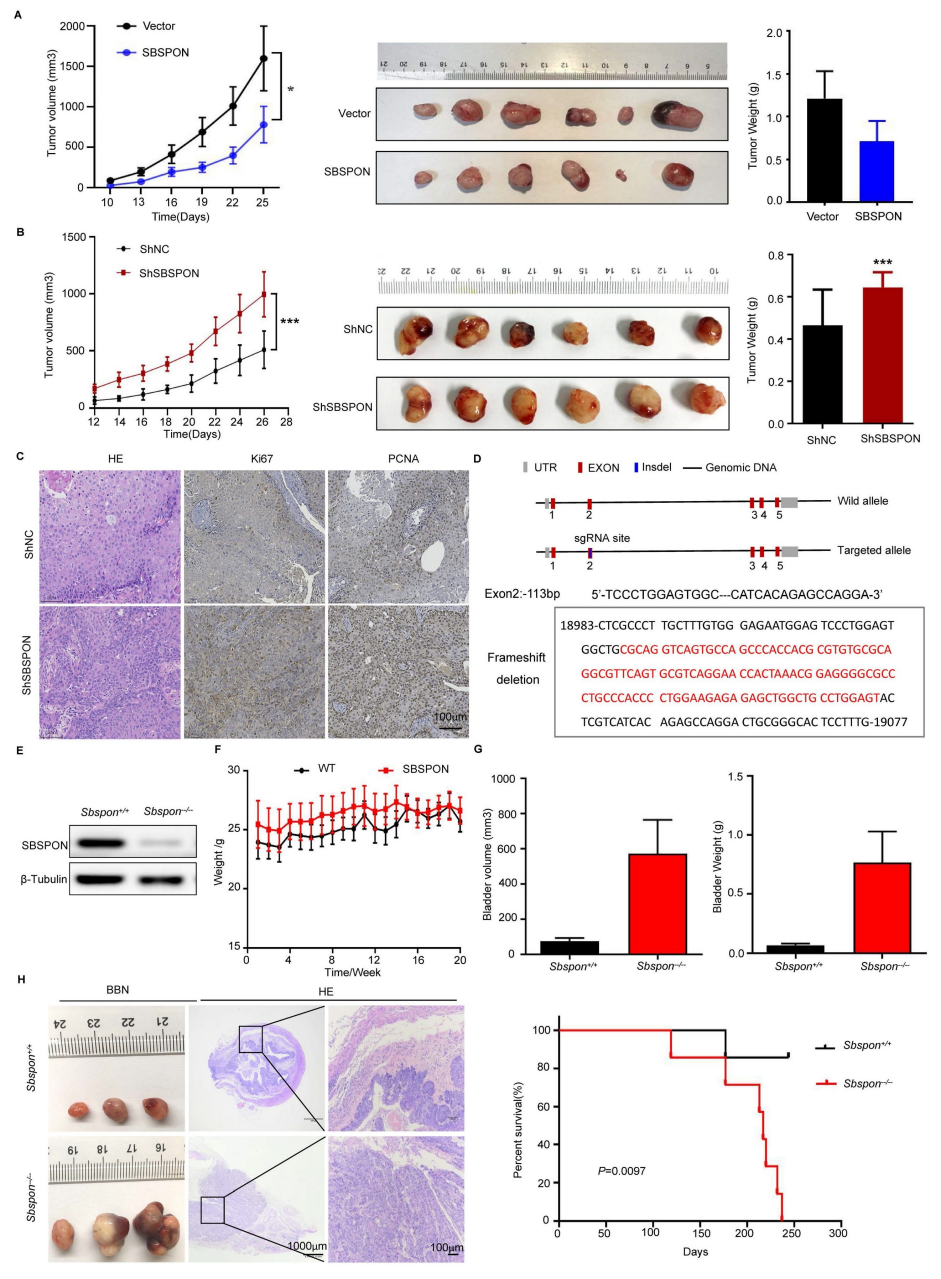
**SBSPON represses* in vivo* bladder cancer tumorigenesis.** (A, B) Subcutaneous injection of indicated SW780 cells into mice. Tumor images, tumor weight, and changes in tumor volume during treatment for each treatment group were presented. (C)Immunohistochemical staining exhibited higher Ki67 and PCNA expression levels in the ShSBSPON group. (D) Schematic representation of the strategy used for genetic ablation of *Sbspon* by the CRISPR/Cas9 system. Sanger sequencing confirmed that the introduction of Cas9 and sgRNA induced several deletions, including the frameshift deletions at -113 base pairs. (E)Western blot analysis showed negative expression of Sbspon in *Sbspon^-/-^* mice. (F) No significant differences were observed in body weight changes over time between *Sbspon^-/-^* and their wild-type counterparts (*Sbspon^+/+^*) mice. (G) The volume and weight of bladder tumors in mice treated with BBN were measured. (H) Representative images of bladder tissues from *Sbspon^-/-^* or wild-type mice after 18 weeks of treatment. Kaplan-Meier curves for the overall survival of *Sbspon^-/-^* and wild type mice treated with BBN for 237 days. *P < 0.05, ***P < 0.001.

To investigate the effect of SBSPON on the capacity of tumor growth *in vivo*, we established stable SBSPON-overexpressing and SBSPON-knockdown cell lines in SW780 cells by constructing lentiviral vectors. Male BALB/c nude mice were subcutaneously injected with bladder cancer cells. The results showed that overexpression of SBSPON suppressed tumor growth (Fig. 4A). Conversely, SBSPON-knockdown (ShSBSPON) exhibited enhanced tumorigenic capacity relative to the ShNC control (Fig. 4B). As shown in Fig. 4C, immunohistochemical analysis revealed that SBSPON knockdown increased the expression of proliferation markers Ki67 and PCNA compared to the ShNC control. Taken together, these results provide strong evidence that SBSPON inhibits the tumorigenesis of bladder cancer.

To further confirm the* in vivo* physiological role of SBSPON on bladder tumorigenesis, we generated *Sbspon* knockout mice by introducing a frameshift deletion in *Sbspon* via the CRISPR/Cas9 system. We designed two sgRNAs specifically targeting exon 2 in mouse *Sbspon* (Fig. 4D) and cloned them into the pUC57-T7-gRNA vector. After co-injecting Cas9 mRNA and the sgRNAs into C57BL/6J mouse embryos, 19 pups from 5 litters were recovered and we obtained 5 founder individuals. Sanger sequencing confirmed that the induction of Cas9 and sgRNAs induced several deletions, including the frameshift deletions at -113 base pairs. We obtained F1 generation *Sbspon* knockout mice by breeding the founder mice with C57BL/6J mice, and then generated homozygous *Sbspon* knockout mice by inter-crossing F1 heterozygous mice. The significantly reduced Sbspon protein in the knockout mice was confirmed by western blot (Fig. 4E). While *Sbspon^-/-^* female mice did not exhibit any observable phenotypic differences, *Sbspon^-/-^* male mice exhibited increased grip strength, higher alanine aminotransferase, and lower total bilirubin in serum (Fig. S4).

Given decreased SBSPON expression in bladder cancer patients and bladder cancer cells, we investigated the possibility that loss of *Sbspon* might accelerate tumor growth and progression in mice. BBN was used to induce bladder cancer. The results showed that there are no significant differences in body weight changes over time between *Sbspon^-/-^* mice and their wild-type counterparts (*Sbspon^+/+^*) (Fig. 4F). Notably, the volume of bladder tumors and the total tumor burden were higher in *Sbspon^-/-^* mice, compared to *Sbspon*^+/+^ mice (Fig. 4G). Hematoxylin-eosin (HE) staining showed that the bladder cancer cells were more condensed and that the size of the nuclei was larger in tumors from *Sbspon^-/-^* mice than in tumors from *Sbspon*^+/+^ mice (Fig. 4H). Furthermore, we induced *Sbspon^-/-^* mice and *Sbspon*^+/+^ mice with BBN for a period and observed the survival time of the mice (n=7 per group). The median survival time for *Sbspon^-/-^* mice was 217 days and the maximum survival time was 237 days, while only one *Sbspon*^+/+^ mouse died, at 177 days, indicating that the loss of *Sbspon* negatively correlates with survival in our cancer model (Fig. 4H). Thus, genetic ablation of *Sbspon* significantly accelerates the progression of BBN-induced bladder cancer.

### SBSPON is a novel HSPA5 binding glycoprotein

**Figure 5:**
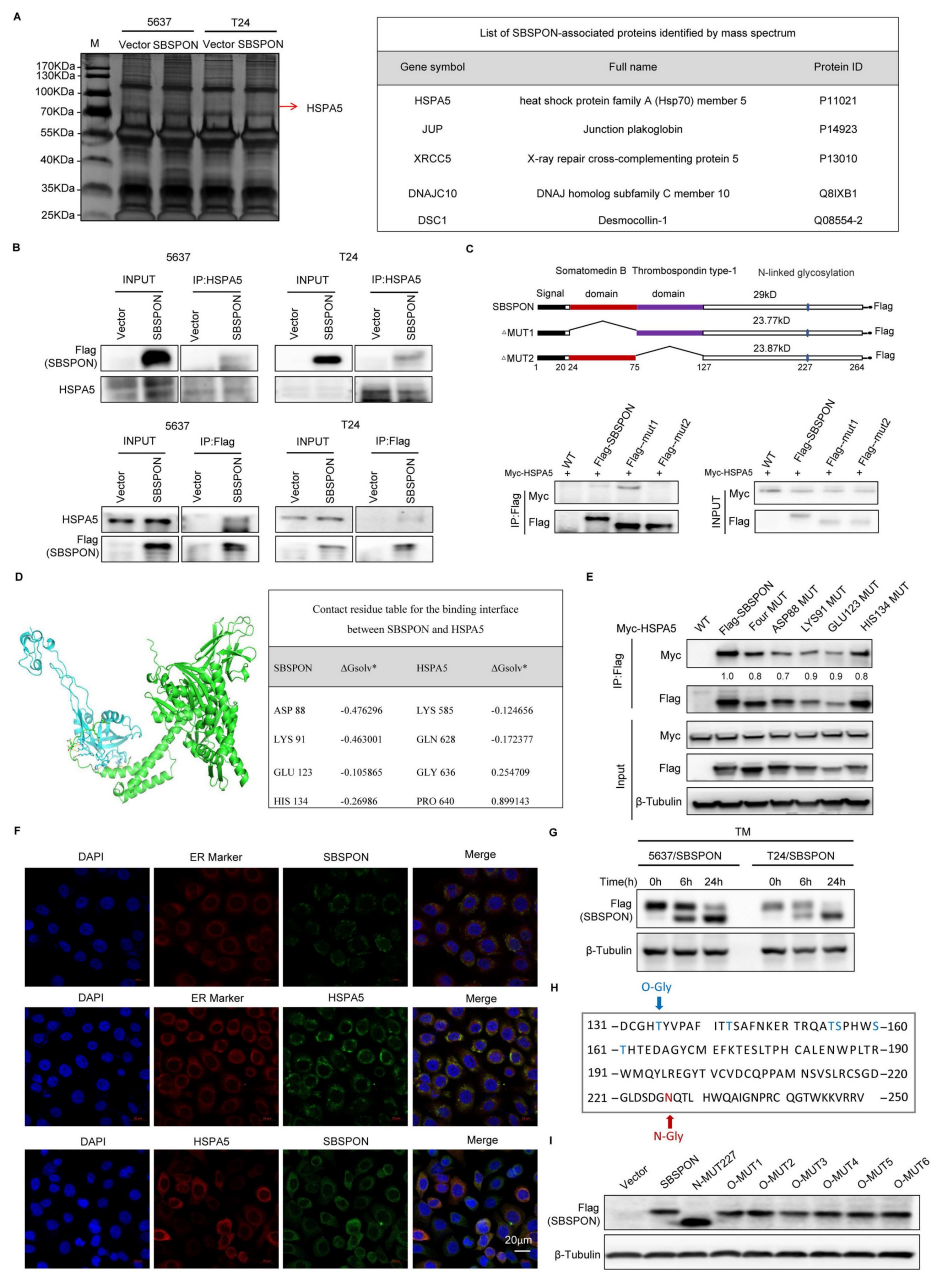
**SBSPON specifically interacts with HSPA5 in bladder cancer.** (A) Immunoprecipitation, followed by mass spectrometry analysis, identified HSPA5 as a highly specific SBSPON-interacting proteins in 5637 and T24 cells. (B) The interactions of SBSPON with HSPA5 were determined by a Co-Immunoprecipitation assay. (C) Schematic representation of full-length and truncated Flag-tagged SBSPON. Western blot analysis was performed to examine the relationship between truncated SBSPON and full-length HSPA5. (D) Hydrogen bonding interactions between SBSPON (cyan) and HSPA5(green). (E)Co-IP assays were performed to identify critical binding residues between SBSPON and HSPA5. (F) Colocalization of SBSPON and HSPA5 in the endoplasmic reticulum was determined by double immunofluorescence staining for SBSPON and HSPA5 in bladder cancer cells. (G) The removal of glycan chains using TM increased the ratio of low molecular weight SBSPON protein. (H) SBSPON has seven specific glycosylation sites, including six O-glycosylation sites and one N-glycosylation site. (I) Only mutated SBSPON (Asn227) displayed lower molecular weight than wild-type SBSPON. β-Tubulin was utilized as a loading control.

To further explore the downstream mechanisms of SBSPON, we carried out immunoprecipitation in SBSPON-3×Flag overexpressing 5637 and T24 cells and proteomic mass spectrometry analysis was performed. HSPA5, DNAJC10, XRCC5, JUP and DSC1 were identified as putative SBSPON binding proteins (Fig. 5A, Table S1). Among these, both HSPA5 and DNAJC10 belong to heat shock family and are ER-associated action proteins^10^. HSPA5 is a key regulator of the UPR and has significant implications in cell survival and tumor progression, while reports on the roles of DNAJC10 in tumorigenesis are limited^33^-^36^. Therefore, HSPA5 was selected as a possible SBSPON binding molecule.

The HSPA5-SBSPON interaction was validated through reciprocal Co-Immunoprecipitation (Co-IP) assays. Cell lysates were immunoprecipitated with anti-Flag (SBSPON) or anti-Myc (HSPA5) antibodies, followed by immunoblotting of the Co-IP complex (Fig. 5B). In addition, bioinformatics analysis indicated that SBSPON contained a somatomedin B domain and a thrombospondin type-1 domain. To determine the SBSPON domains involved in the HSPA5 interaction, we generated two deletion mutants of Flag-tagged SBSPON. Co-IP revealed that the thrombospondin type-1 domain of SBSPON (amino acids 75-127) was required for the interaction between HSPA5 and SBSPON (Fig. 5C).

To identify the specific binding regions mediating SBSPON-HSPA5 interaction, we predicted key binding residues via PPI (Protein Protein Interaction) analysis using the sequences of protein SBSPON (residues 21-264) and protein HSPA5 (residues 19-654) (Fig. 5D, Fig. S5). Critical domains (SBSPON's) are prioritized for mutagenesis. Based on predictions, we will generate mutants for each and all of four amino acid residues (ASP88Vla, LYS91Vla, GLU123Vla, HIS134Vla) (alanine scanning). The results showed the binding of SBSPON to HSPA5 was reduced when ASP88 was mutated as Vla by using Co-IP (Fig. 5E). These data showed that ASP88 is the key binding site of SBSPON to HSPA5.

Confocal microscopy images showed that SBSPON was localized to the cytoplasm near the nuclear membrane. Additionally, co-staining with the ER-Tracker Red revealed that SBSPON was mainly located in the ER (Fig. 5F). Notably, a significant proportion of colocalization pixels, indicating the interplay between HSPA5 and SBSPON, coincided with the regions labeled by the ER tracker. These findings strongly suggest that there is an interaction between HSPA5 and SBSPON within the ER.

Thus, we speculate that SBSPON may be involved in the ER stress response. To test this hypothesis, we evaluated the effect of tunicamycin (TM)-induced ER stress on the expression of SBSPON. As expected, we observed a time-dependent up-regulation of HSPA5 after TM treatment (Fig. S6A). However, we observed the reduction of SBSPON protein level and a novel 30kD protein band, the predicted molecular mass of SBSPON (Fig. 5G).

Considering TM is a protein N-glycosylation inhibitor. This suggests that SBSPON may be a glycoprotein. The Universal Protein Resource (UniProt) database predicts that SBSPON has seven potential specific glycosylation sites, including six O-glycosylation sites (Thr135, Thr143, Thr155, Ser156, Ser160 and Thr161) and one N-glycosylation site (Asn227) (Fig. 5H). To investigate the glycosylation sites of SBSPON protein, we introduced point mutations in the seven potential glycosylation sites. Among these mutations, only the mutation at Asn227 resulted in a lower molecular weight compared to the wild-type SBSPON protein in bladder cancer, suggesting that the potential of glycosylation modification at the Asn227 site (Fig. 5I). Collectively, we identified that SBSPON was a novel glycoprotein.

### SBSPON suppresses AKT/GSK-3β/β-catenin signaling cascade through inhibiting HSPA5 membrane translocation

**Figure 6:**
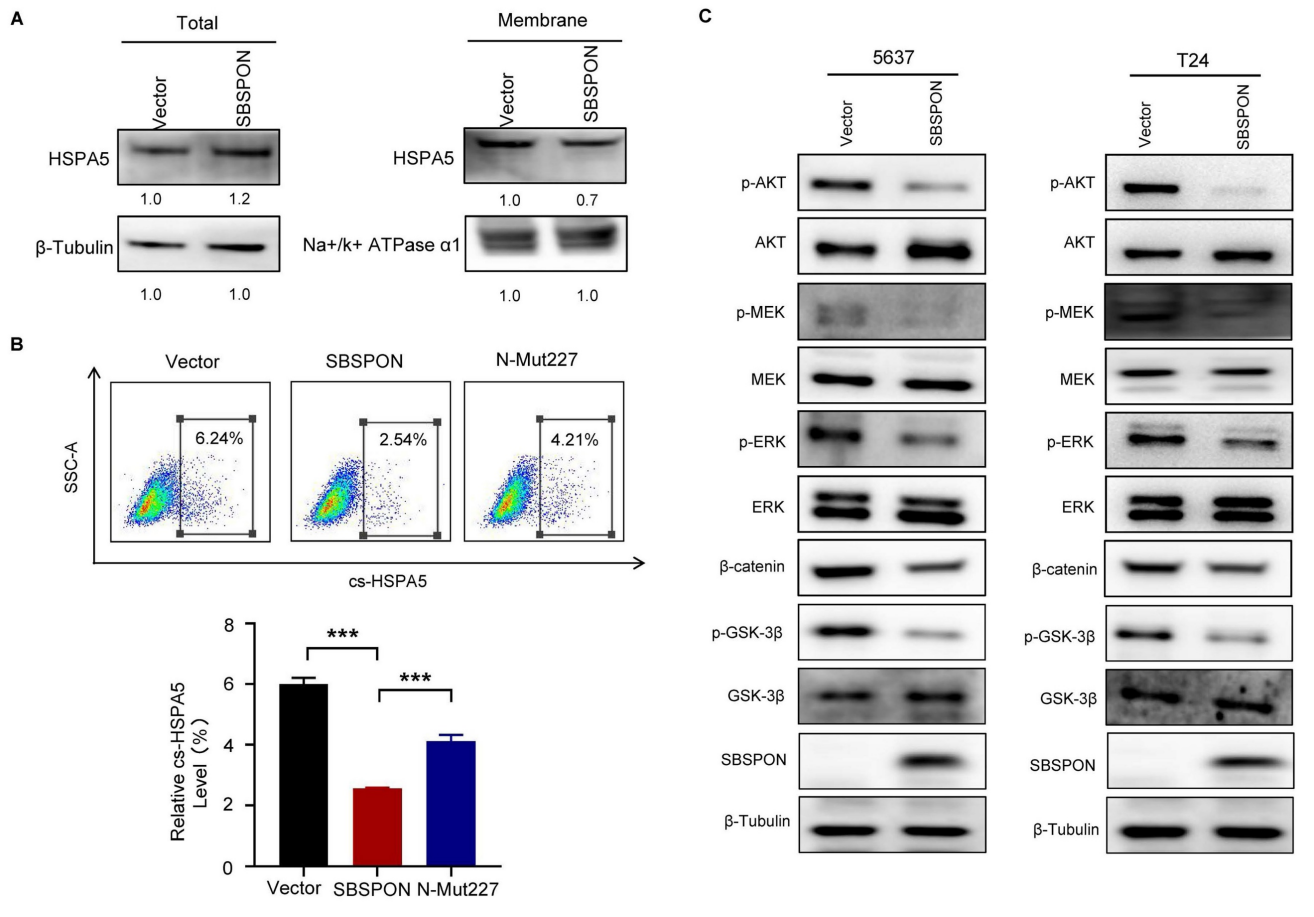
** SBSPON suppresses AKT/GSK-3β/β-catenin signaling cascade through inhibiting HSPA5 membrane translocation.** (A) Western blot was conducted to detect HSPA5 in whole cell lysate and membrane fractions of 5637 cells. Na^+^/K^+^ ATPase α1 and β-Tubulin were used as markers for the membrane and the whole cell lysate fractions, respectively. (B) The cell surface translocation of HSPA5 was detected by flowcytometry assay. ***P < 0.001. (C) Western blot analysis was performed to assess the levels of AKT, ERK, MEK and GSK-3β, and their phosphorylation in 5637 and T24 cells. β-Tubulin was utilized as a loading control.

It was reported that HSPA5 functioned as an oncogene through translocating to the cell surface (csHSPA5)^14^-^19^. Thus, we investigated whether SBSPON functioned through inhibiting HSPA5 membrane translocation. We determined the effects of SBSPON on HSPA5 location. The results showed that SBSPON-overexpressing reduced HSPA5 protein level in the membrane fraction in bladder cancer cells (Fig. 6A). Consistent with this, bladder cancer cells with csHSPA5 were significantly reduced in SBSPON-overexpressing cells compared with that in the control, as determined by flow cytometry (Fig. 6B). Interestingly, we found that the mutation of N-glycosylation on Asn227 of SBSPON (Mut-227) could partly rescue the level of cancer cells with csHSPA5(Fig. 6B). These results indicate that SBSPON can inhibit HSPA5 membrane translocation, with the glycosylation of SBSPON potentially playing a crucial role in the process.

It was reported that csHSPA5 can promote tumor progression through activating PI3K/AKT, MAPK/ERK and GSK-3β/β-catenin signaling cascade in several cancers^14^-^19^. Therefore, we analyzed the effects of SBSPON overexpression on several csHSPA5-related pathways in bladder cancer cells. The results showed that SBSPON overexpression resulted in a notable reduction in the levels of phosphorylated AKT (p-AKT S473), phosphorylated-extracellular signal-regulated kinase 1/2 (p-ERK) and phosphorylated-mitogen-activated protein kinase (p-MEK) while it had no effect on the total AKT, ERK and MEK proteins (Fig. 6C). Addition, SBSPON overexpression resulted in a significant inhibition of phosphorylated GSK-3β and β-catenin protein while GSK-3β protein level was slightly increased (Fig. 6C). These results suggest that SBSPON functions as a tumor suppressor by modulating the AKT/GSK-3β/β-catenin signaling pathway.

### SBSPON inhibits the resistance of bladder cancer cells to cisplatin via interrupting the binding between HSPA5 and PERK

**Figure 7:**
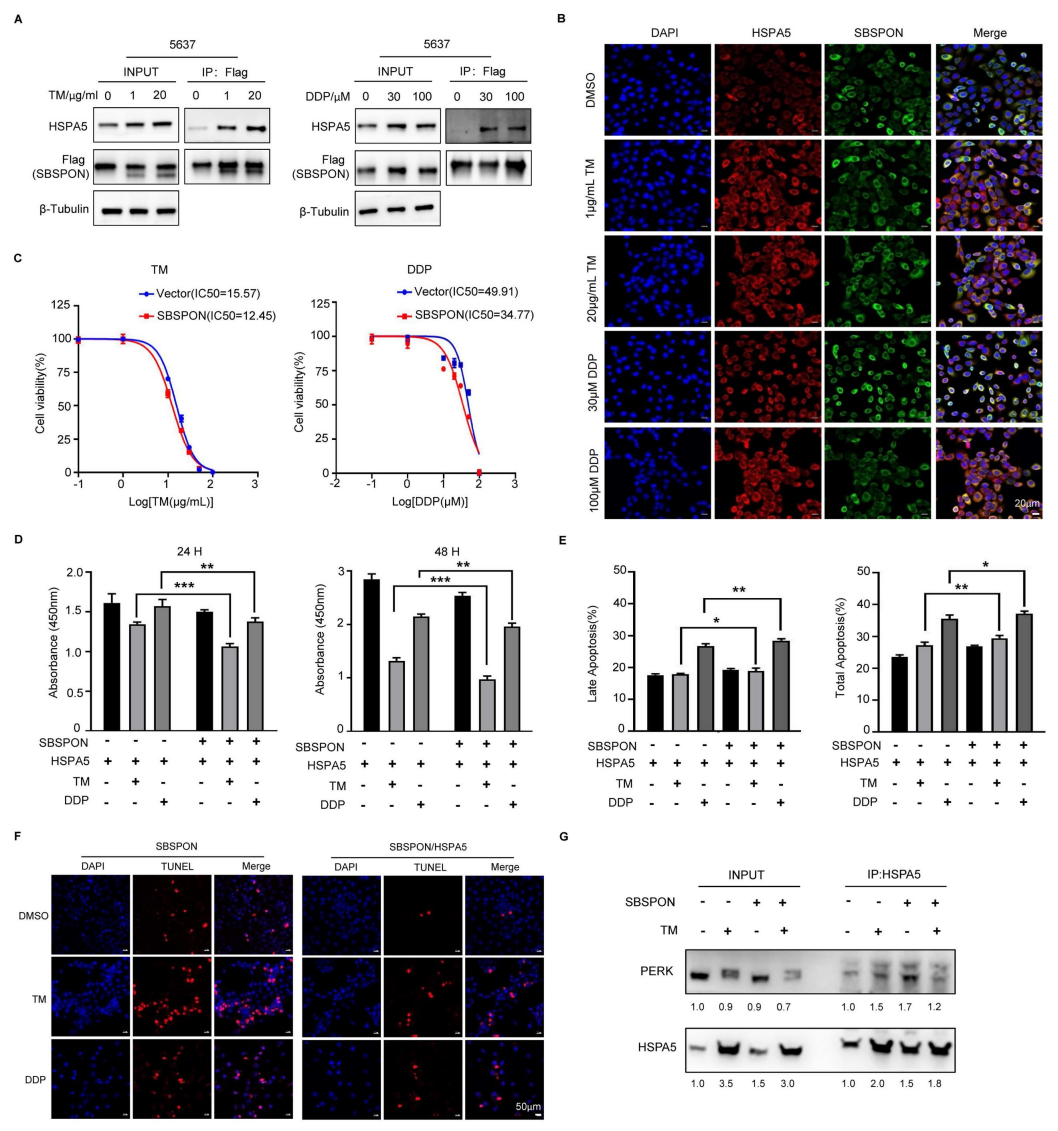
** SBSPON inhibits the resistance of bladder cancer cells to cisplatin through reducing the inhibitory effect of HSPA5 on apoptosis.** (A) The interaction between SBSPON and HSPA5 increased under ER-stress exposed to DDP and TM. β-Tubulin was utilized as a loading control. (B) A greater concentration of colocalized pixels was seen in the interaction between these two proteins following induction of ER stress with DDP or TM exposure. (C)The IC50 value of DDP in SBSPON overexpressing cells was decreased. (D) The cell viability of SBSPON in HSPA5-overexpression cells was significantly decreased compared to the HSPA5-overexpression cells following exposure to DDP and TM exposure. (E) Cell apoptosis of SBSPON in HSPA5-overexpression cells was significantly increased compared to the HSPA5-overexpression cells following exposure to DDP and TM exposure. (F) TUNEL staining was carried out to analyze SBSPON-overexpression and SBSPON/HSPA5-overexpression cells apoptosis. (G) The interplay between HSPA5 and PERK was observed to be reduced in SBSPON-overexpression cells subsequent to their exposure to TM. *P < 0.05, **P < 0.01, ***P < 0.001.

The above results indicated that SBSPON interacted with HSPA5 in bladder cancer. As a ER stress-response protein, HSPA5 plays a key role in regulating ER stress processes that are closely related to drug resistance of tumor cells^23^, ^25^. We investigated whether SBSPON was involved with drug resistance of tumor cells under ER stress. We investigated the interaction between SBSPON and HSPA5 under ER stress. The results showed that interaction between SBSPON and HSPA5 was enhanced upon ER stress induced by DDP and TM (Fig. 7A), which was confirmed by immunofluorescence co-localization (Fig. 7B). We then investigated the effect of SBSPON on the chemosensitivity of bladder cancer cells to DDP using CCK-8 assays. The overexpression SBSPON significantly decreased the IC50 of DDP in bladder cancer cells compared with the control, while slightly reduced the IC50 of TM (Fig. 7C). These findings suggest that the interplay between SBSPON and HSPA5 is dynamic and can be influenced by cellular stress like exposure to chemotherapy.

We evaluated the impact of SBSPON and HSPA5 expression levels on cell viability and apoptosis under ER stress conditions, including chemotherapy. The overexpression of SBSPON can significantly counteract the growth promotion and the inhibition of apoptosis of HSPA5 overexpression on bladder cancer cells when treated with DDP and TM (Fig. 7D-E), while the overexpression of HSPA5 had the opposite effects on bladder cancer cells with overexpressed SBSPON (Fig. S7A).

Furthermore, we analyzed cell apoptosis in the same experimental group using TUNEL assays. The number of TUNEL-positive apoptotic nuclei was significantly reduced in SBSPON/HSPA5-overexpression bladder cancer cells compared to the SBSPON overexpression cells (Fig.7F, Fig. S7B). And a more robust reduction of apoptosis in the HSPA5/SBSPON-overexpression cells compared to the SBSPON-overexpression cells when treated with DDP (Fig. 7F, Fig. S7B).

To provide more convincing evidence unraveling that SBSPON is involved with drug resistance of tumor cells through binding to HSPA5 under ER stress, we performed Co-Immunoprecipitation between HSPA5 and PERK. The results showed that the level of interplay between HSPA5 and PERK was observed to be reduced in SBSPON-overexpression cells after their exposure to TM (Fig. 7G). PERK disassociation from HSPA5 subsequently initiated ER stress-mediated apoptosis. These findings suggested that SBSPON overexpression triggered ER stress-mediated apoptosis via interrupting the binding between HSPA5 and PERK.

Overall, this study highlighted the significant finding that SBSPON could modulate ER stress signaling by interacting with HSPA5 and further enhanced apoptosis following chemotherapy in bladder cancer by promoting ER stress-induced cell death. These findings shed light on the potential of SBSPON as a therapeutic target to improve the chemotherapy efficacy for bladder cancer.

## Discussion

In the present study, we identified SBSPON as a tumor suppressor in bladder cancer. Our data show that (a) SBSPON is markedly downregulated in human bladder cancer, (b) SBSPON acts as a potential tumor suppressor *in vivo* and *in vitro*, (c) SBSPON is a novel HSPA5 binding glycoprotein, (d) SBSPON inhibits bladder cancer process through HSPA5/AKT/GSK-3β signal pathway, (e) SBSPON inhibits cisplatin resistance of bladder cancer cells by attenuating HSPA5's apoptotic inhibitory effects through physically interacting with HSPA5. Thus, these results reveal a potential mechanism and the clinical significance of glycoprotein SBSPON in bladder cancer.

In our investigation, we have substantiated low expression of SBSPON in bladder cancer through comprehensive data mining and two independent internal cohort studies, yielding robust and dependable outcomes. We found that SBSPON downregulation in bladder cancer is closely associated with tumor clinical staging, pathological grade, regional lymph node metastases, and bladder cancer patients with low SBSPON expression have a worse prognosis survival prognosis (Fig. 1 and Table 1). We have demonstrated that SBSPON significantly inhibits i*n vitro* bladder cancer cell growth through induction of G0/G1 cell cycle arrest, and induces apoptosis of bladder cancer cells (Fig. 2). We revealed that SBSPON regulates the expression of EMT-related markers, such as E-cadherin, N-cadherin, Vimentin and Snail, leading to reduced migration and invasion of bladder cancer cells (Fig. 3).

In addition, SBSPON knockdown significantly increase tumor volume in an *in vivo* xenograft model of bladder cancer (Fig. 4A-B). Genetic ablation of *Sbspon* cannot induce spontaneous bladder cancer, but significantly accelerates the progression of BBN-induced bladder cancer (Fig. 4C-G). BBN is a chemical carcinogen of the bladder. Chronic exposure to BBN induces bladder cancer in rodent models through DNA adduct formation in the urothelium^37^-^39^, which has similarities in both molecular biology and histology^37^, ^40^, ^41^. It is reported that specific gut microbiome can metabolize BBN, thereby contributing factors for chemical-induced carcinogenesis. Our data suggest that SBSPON may suppress the metabolizing BBN, which could open avenues to improve predisposition risk assessment and prevention of bladder cancer. These findings* in vitro* and *in vivo* reveal a close association between SBSPON and the occurrence and development of bladder cancer.

In this study, we identified SBSPON as a novel HSPA5-binding glycoprotein (Fig. 5). First, our findings showed an interaction between HSPA5 and SBSPON within the ER. HSPA5 locates in the ER and mainly functions as a chaperone heat shock protein. However, we observed the reduction of SBSPON protein level and a novel 30kD protein band in the predicted molecular mass of SBSPON by TM-induced ER stress, suggesting that SBSPON may be a glycoprotein (Fig. 5E). Glycosylation is the most complex and most abundant post-translational modification. Protein glycosylation includes two major categories, N-linked and O-linked glycosylation^42^. We determined one N-glycosylation site (Asn227) in seven glycosylation sites predicted in SBSPON protein (Fig. 5E-G). We do not know if glycosylation of SBSPON involves the help of further UPR proteins by binding HSPA5. We observed that the glycosylation of SBSPON can slightly decelerate the HSPA5 turnover and accelerate slightly SBSPON turnover (Fig. S6B), and especially inhibit HSPA5 membrane translocation (Fig. 6A-B).

It was reported that HSPA5 is upregulated in bladder cancer tissues and functions as oncogene in bladder cancer^12^. HSPA5 is normally retained in the ER by its KDEL motif, but in the progression of cancer, excess HSPA5 in the ER is translocated to the cell surface (csHSPA5) that stimulates the PI3K/AKT pathways, promoting promote cell survival and inhibit apoptosis^14^-^19^. Our data revealed that the overexpression of SBSPON can slightly increase the amount of HSPA5 protein (Fig. S6C), which seems to contradict the anti-cancer function of SBSPON. However, we found that SBSPON can inhibit HSPA5 membrane translocation (csHSPA5) (Fig 6A-B) and reduce p-AKT, p-GSK-3β and β-catenin protein (Fig. 6C), which is consistent with previous reports^43^-^45^. These results suggest that SBSPON functions as a tumor suppressor by modulating the AKT/GSK-3β/β-catenin signaling pathway. Thus, the tumor-suppressive effects of SBSPON, at least partially, were achieved through suppressing the phosphorylation of AKT by inhibiting the membrane translocation of HSPA5 (Fig. 6), thereby inhibiting HSPA5's promoting effect on tumors. The mechanism could potentially explain the observed contradict.

Chemotherapy, especially with DDP, is a primary treatment for bladder cancer; however, resistance to this drug frequently results in unfavorable patient outcomes^46^-^49^. ER stress has been associated with chemotherapy resistance in various cancers^50^-^54^. HSPA5 plays a critical role in ER stress signaling and promotes cell survival under stress by interacting with misfolded proteins, making it pivotal in modulating cellular sensitivity to DDP in bladder cancer^51^, ^52^, ^54^. In this study, we found that interaction between SBSPON and HSPA5 was enhanced upon ER stress induced by DDP (Fig. 7A-B). Furthermore, SBSPON could attenuate HSPA5's inhibitory effect on ER stress-mediated cell death in bladder cancer after DDP treatment (Fig. 7D-F). These findings shed light on that SBSPON could inhibit cisplatin resistance of bladder cancer by interacting with HSPA5.

It has been reported that PERK, one of the most important ER membrane proteins, can play an important role in the processes of tumor, including EMT and apoptosis^55^. Under prolonged or severe stress conditions, the dissociation of HSPA5 from the ER stress sensor PERK occurs, resulting in cellular apoptosis through activating PERK/eIF2α/CHOP pathway^50^, ^53^. Considering the cellular location of SBSPON, we investigated the interaction between HSPA5 and ER sensors PERK in bladder cancer cells using a Co-IP assay. Interestingly, we found that SBSPON had the potential to attenuate the interaction between HSPA5 and PERK, resulting in a lowered threshold for the activation of ER stress signaling. In addition, our results showed that CHOP was induced in SBSPON-overexpression cells following administration of DDP and TM (Fig. S7C). The transcription factor CHOP is recognized for its pivotal role in mediating apoptosis triggered by ER stress. Thus, we concluded that SBSPON overexpression triggered ER stress-mediated apoptosis via interrupting the binding between HSPA5 and PERK.

However, there are still some limitations to this study. The bladder cancer tissues with survival information used in study are limited. Whether SBSPON involves in UPR by binding both HSPA5 and DNAJC10 is not investigated. In future studies, we will do further investigation.

## Conclusions

**Figure 8:**
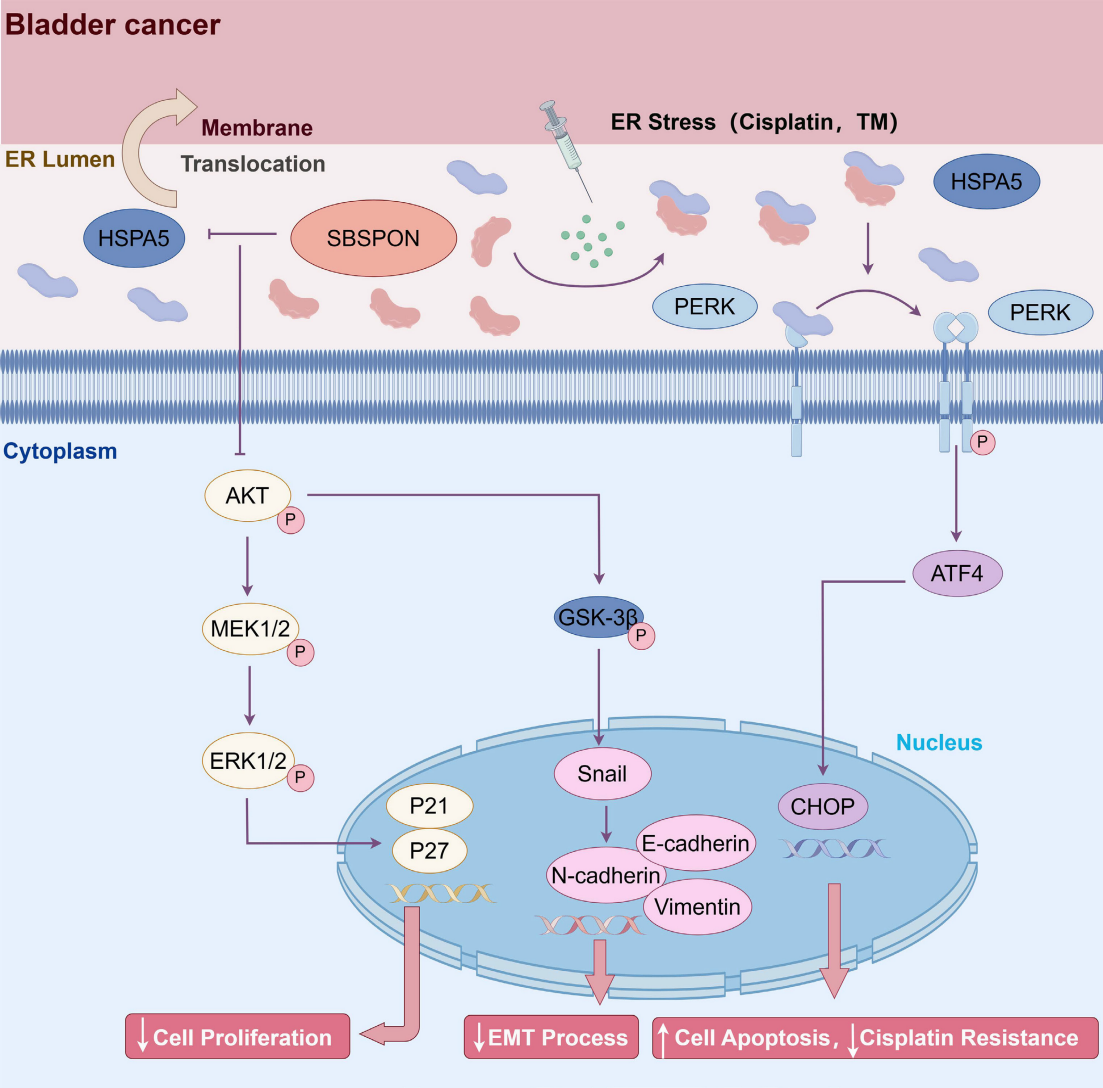
**Schematic representation of SBSPON action in bladder cancer cells.** SBSPON inhibits HSPA5 membrane translocation, ultimately suppressing bladder cancer cell proliferation via inhibition of the HSPA5-AKT-MEK/ERK pathway. SBSPON may inhibit EMT process through GSK-3β/Snail signal pathway. A novel interaction between HSPA5 and SBSPON enhanced following treatment of ER stress inducers DDP and TM. SBSPON can attenuate the binding between HSPA5 and PERK, leading to a lower threshold for activation of ER stress signaling and ultimately induces cell death associated with ER stress (by Figdraw).

In conclusion, SBSPON was identified as a novel-tumor suppressor molecule for bladder cancer. SBSPON inhibited bladder cancer progress by suppressing the AKT signal pathway through inhibiting HSPA5 membrane translocation. Moreover, SBSPON could modulate ER stress signaling through competitively binding HSPA5 with ER sensors PERK under ER stress and further potentiate apoptosis during chemotherapy treatment for bladder cancer through the induction of ER stress (Fig. 8). Collectively, our study indicated that SBSPON has the potential to serve as a biomarker for bladder cancer, and this may provide novel therapeutic targets for bladder cancer treatment.

## Supplementary Material

Supplementary figures and table.

## Figures and Tables

**Table 1:** Correlation between SBSPON expression and the clinico-pathologic features of patients with bladder cancer

Characteristic	Low SBSPON expression(n=96)	High SBSPON expression(n=110)	P value
Sex			
<=57	24	24	0.59
>57	72	86	
Age			
Male	63	81	0.211
Female	33	29	
T status			
T1-2	62	86	0.03*
T3-4	34	24	
Grade			
G1-2	18	36	0.023*
G3	78	74	
N status			
N0	85	107	0.013*
N1	11	3	

*, P < 0.05

**Table 2:** Univariate and multivariate analyses for overall survival in bladder cancer

Clinico-pathologic variable	Univariate analysis			Multivariate analysis		
	RR^a)^	95.0%CI^b)^	P value	RR	95.0%CI	P value
Sex (male/female)	0.63	0.69-5.05	0.22	-	-	-
Age (>/<50 years)	0.20	0.49-3.03	0.67	-	-	-
Primary tumor stage (T1-2/T3-4)	-0.33	0.34-1.54	0.40	-	-	-
Grade (G1-2/G3)	-0.88	0.17-1.04	0.06	-	-	-
Lymph node status (negative/positive)	-1.56	0.09-0.49	0.0003**	-1.12	0.13-0.81	0.015*
SBSPON (low/high)	1.30	1.63-8.18	0.0016**	0.98	1.12-6.27	0.026*

^a)^RR, Relative risk; ^b)^CI, Confidence interval; *, P < 0.05, **, P < 0.01
