# Supplementary material for: Novel glycoprotein SBSPON suppressed bladder cancer through the AKT signal pathway by inhibiting HSPA5 membrane translocation
Source: Int J Biol Sci. 2025 Jul 11;21(10):4586–603. doi: 10.7150/ijbs.109973 (PMC12320502; doi:10.7150/ijbs.109973)
Supplement: Supplementary file 1 — Supplementary figures and table. [file ijbsv21p4586s1.pdf]

Supporting Material

Novel glycoprotein SBSPON suppressed bladder cancer through the AKT signal pathway by inhibiting HSPA5 membrane translocation

Beibei Ni, Shi Li, Lan Zhao, et al.

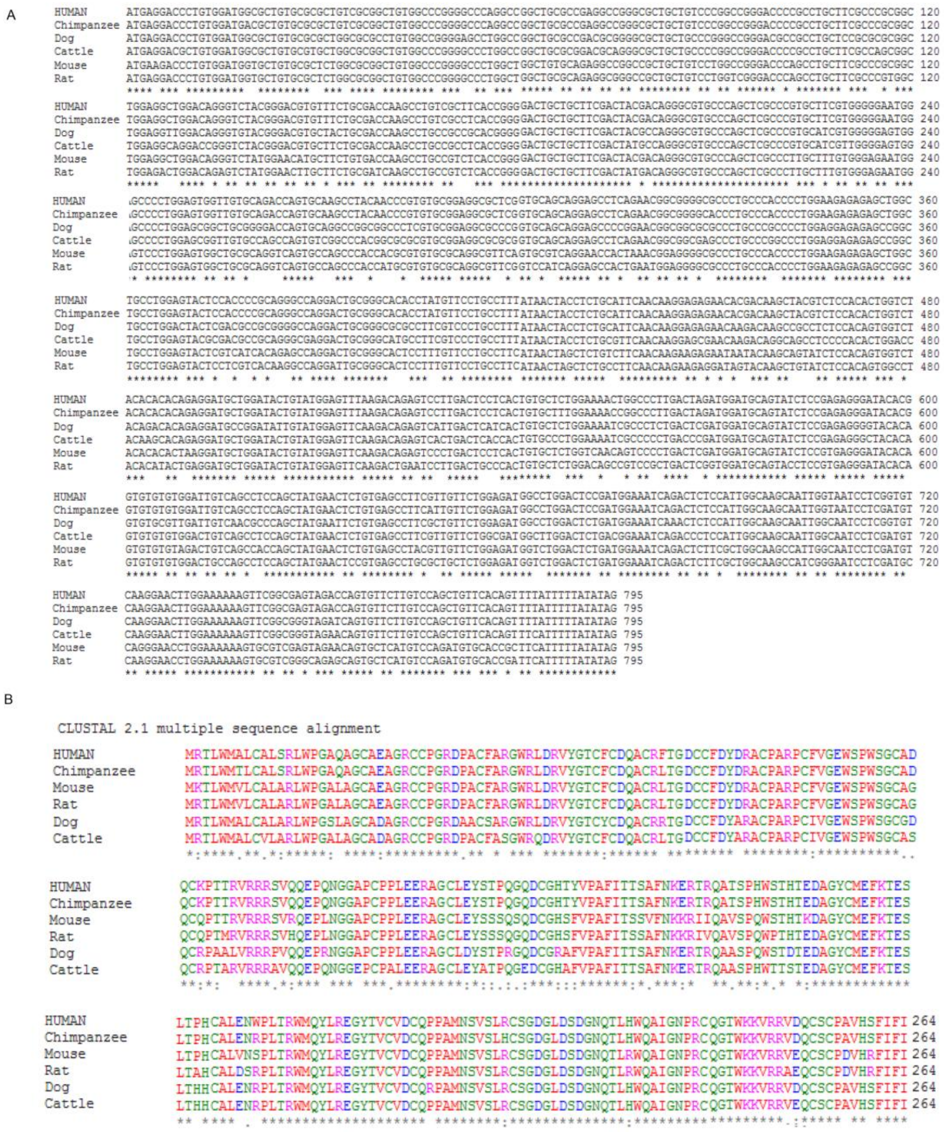

Fig. S1. Sequence alignment of SBSPON orthologs and encoded proteins from human, chimpanzee, dog, cattle, mouse and rat.

(A, B) Sequence alignment of SBSPON orthologs and encoded proteins from human, chimpanzee, dog, cattle, mouse and rat. The gene and protein sequence alignments were initially performed with LaserGene and ClustalW, respectively, and were then manually modified. The conserved (100%) residues are labeled with a star below the residues.

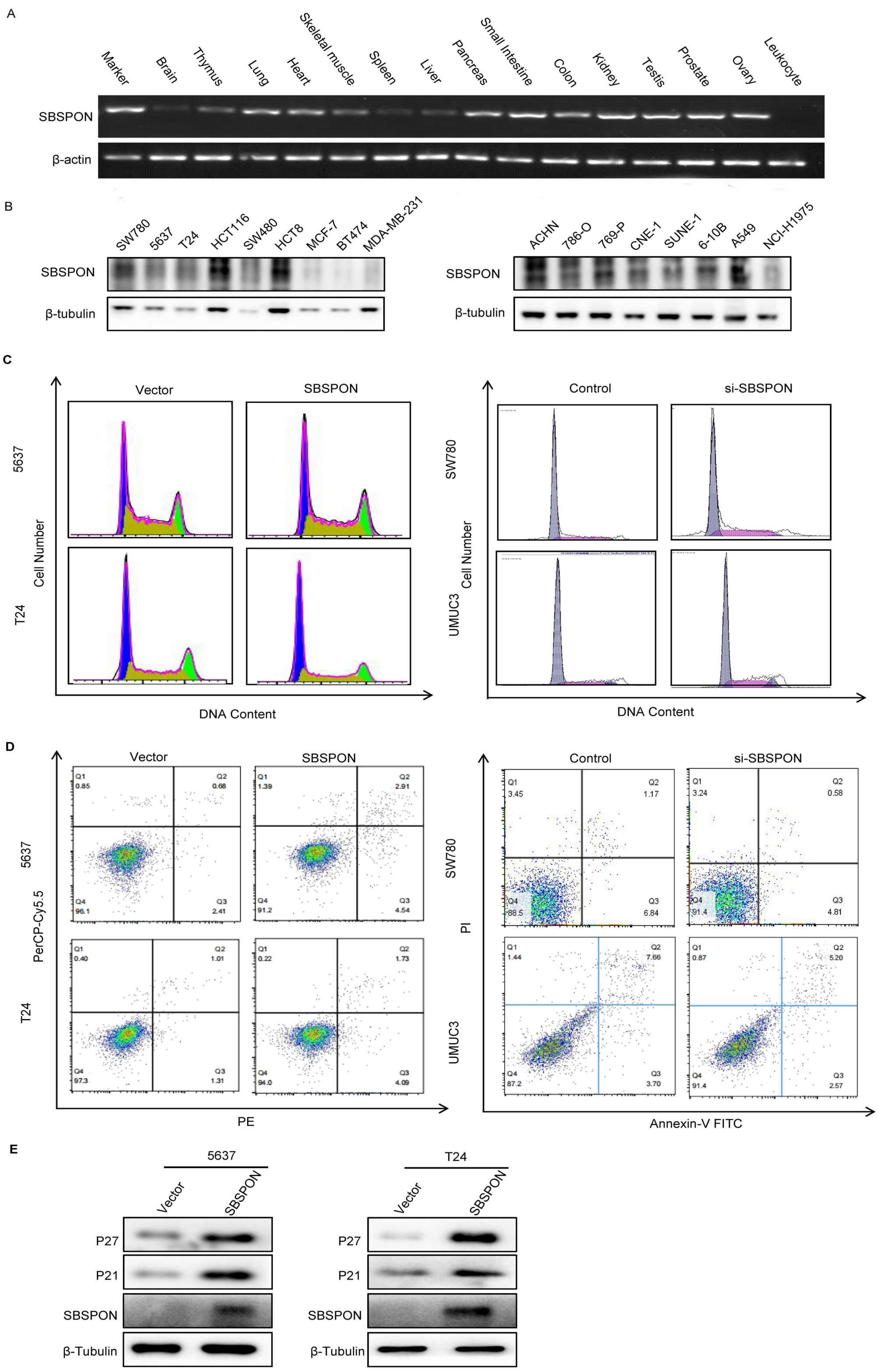

Fig. S2. The profiles and effects of SBSPON on different tissues and cells.

(A) SBSPON mRNA was widely expressed in majority of the normal human tissues

assessed.

(B)Western blot analysis was performed to assess the expression of SBSPON in human tumor cell lines.

(C) The influence of SBSPON on the cell cycle was analyzed using flow cytometry.

(D) The impact of SBSPON on the apoptosis was evaluated using flow cytometry.

(E) The expression levels of P21 and P27 were assessed using western blot analysis. β-Tubulin was utilized as a loading control.

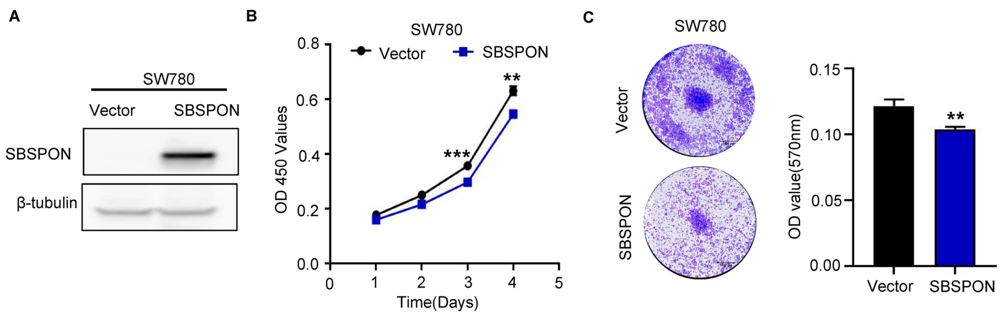

Fig. S3. The effects of SBSPON overexpression on the proliferation and migration were accessed in SW780 cells.

(A) SBSPON expression was determined by western blot analyses following transduction using lentiviral construct. β-Tubulin was utilized as a loading control.

(B) The effect of SBSPON on the proliferation of bladder cancer cells was evaluated using CCK-8 assays.

(C) The impact of SBSPON on the cell migration was accessed by transwell assays.

$^{**}P < 0.01$, $^{***}P < 0.001$.

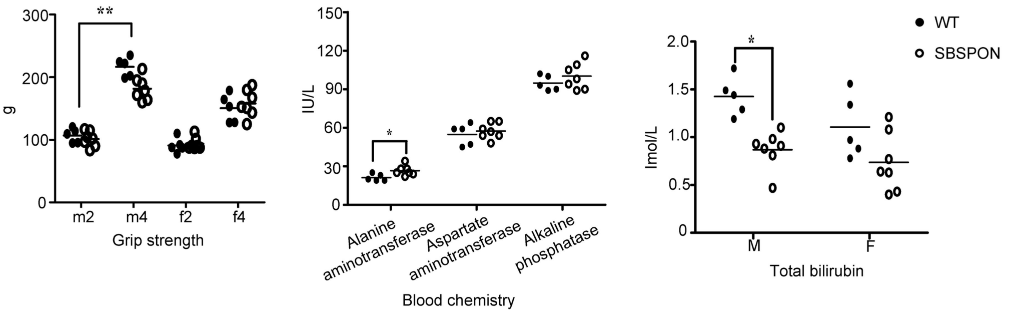

Fig. S4. The phenotypic differences of Sbspon knockout mice and their wild-type counterparts. Sbspon$^{-/-}$ male mice exhibited increased grip strength, higher alanine aminotransferase level, and lower total bilirubin level comparison to wild-type mice. **P* < 0.05, ***P* < 0.01.

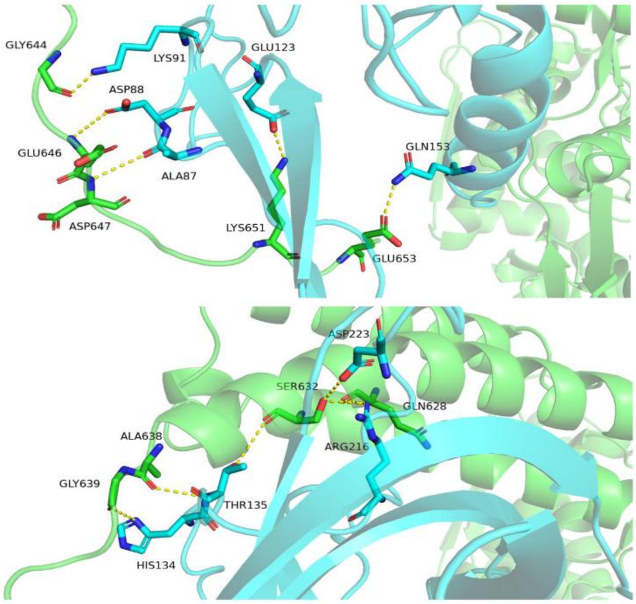

Fig. S5. Hydrogen bonding interactions between SBSPON (cyan) and HSPA5 (green)(Detail).

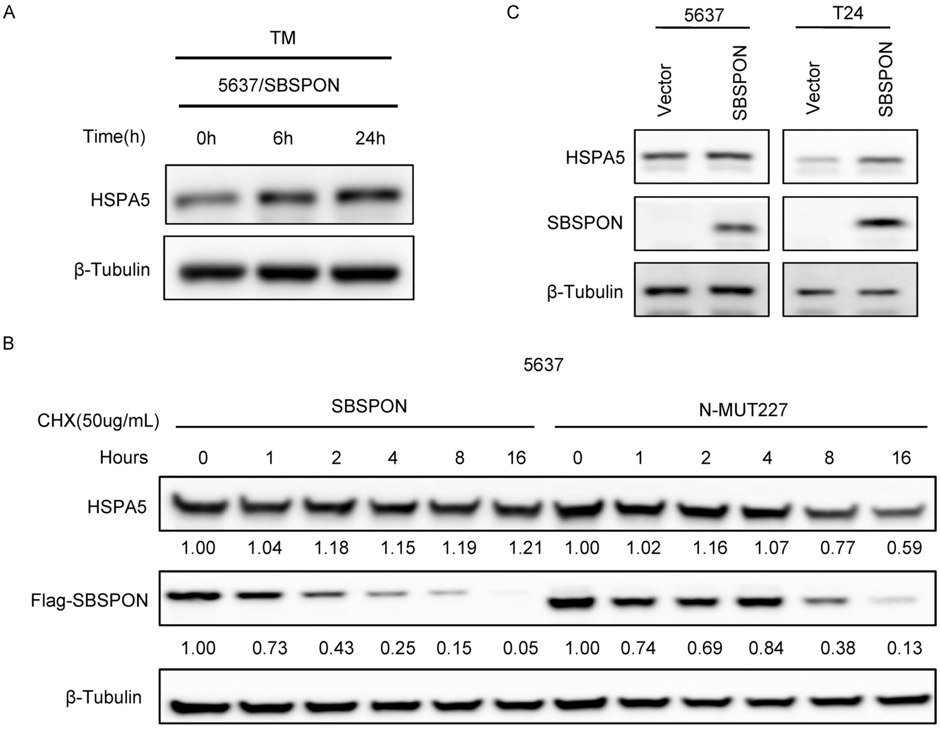

Fig. S6. The expression levels of SBSPON and HSPA5 proteins in bladder cancer cells were assessed under different treatment conditions.

(A) Western blot analysis was performed to assess the expression of HSPA5 after TM treatment.

(B) 5637 cells were treated with CHX, and the SBSPON and HSPA5 protein level was assessed by western blot analysis.

(C) Western blot analysis was performed to assess the levels of HSPA5 in the SBSPON-overexpressing cells. β-Tubulin was utilized as a loading control.

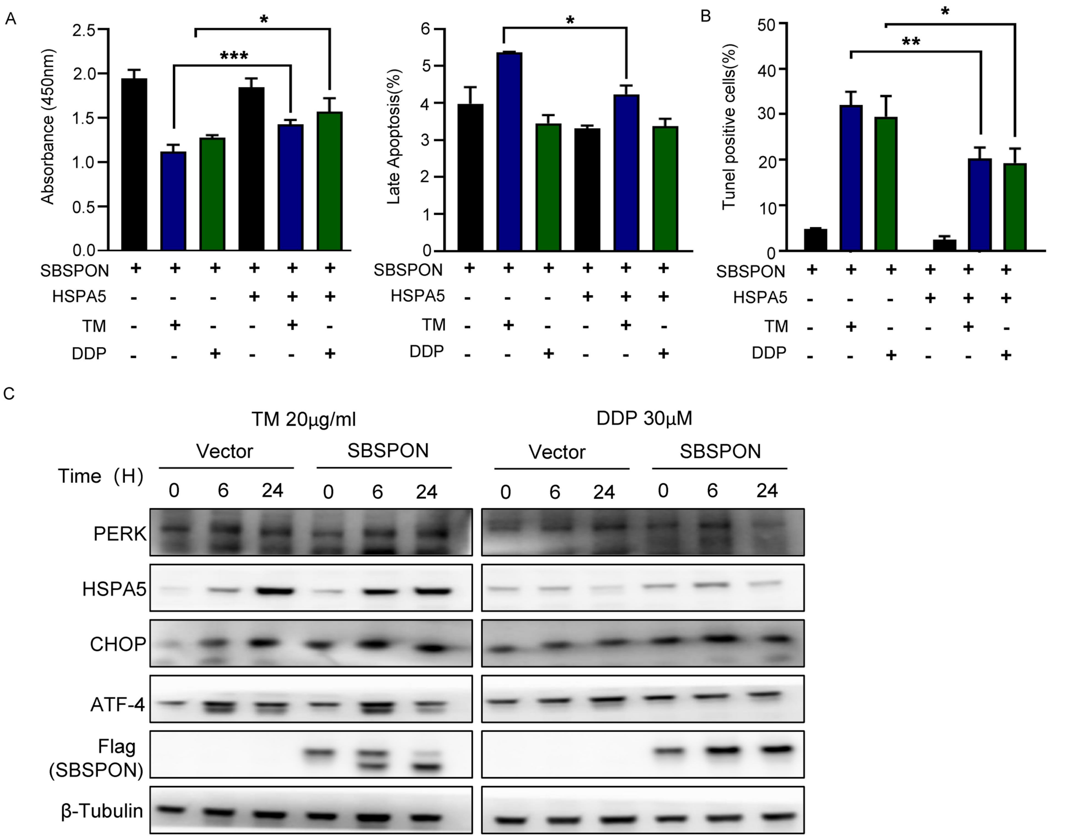

Fig. S7. The functional of SBSPON-overexpression and SBSPON/HSPA5-overexpression cells.

(A) The viability and the apoptosis rate of SBSPON-overexpression and SBSPON/HSPA5-overexpression cells following exposure to DDP or TM.

(B) TUNEL staining was carried out to analyze SBSPON-overexpression and SBSPON/HSPA5-overexpression cells apoptosis.

(C) Western blot analysis was performed to examine the expression of HSPA5, PERK, CHOP and ATF4 in the SBSPON-overexpression cells following exposure to DDP and TM. β-Tubulin was utilized as a loading control.

Table S1 A list of potential SBSPON-interacting protein candidates based on immunoprecipitation assays and mass spectrometry analysis

Name	PepCount	UniquePepCount	Number of proteins	Mol.weight [kDa]
HSPA5	13	12	1	72.332
JUP	8	8	5	81.744
XRCC5	13	13	3	82.704
DNAJC10	20	20	6	91.079
DSC1	5	5	2	93.834
